# Characterization of a Thermostable 8-Oxoguanine DNA Glycosylase Specific for GO/N Mismatches from the Thermoacidophilic Archaeon* Thermoplasma volcanium*

**DOI:** 10.1155/2016/8734894

**Published:** 2016-10-05

**Authors:** Miki Fujii, Chieri Hata, Munetada Ukita, Chie Fukushima, Chihiro Matsuura, Yoshie Kawashima-Ohya, Koji Tomobe, Tsuyoshi Kawashima

**Affiliations:** Department of Molecular Biology, Faculty of Pharmaceutical Science, Yokohama University of Pharmacy, 601 Matano-cho, Totsuka-ku, Yokohama 245-0066, Japan

## Abstract

The oxidation of guanine (G) to 7,8-dihydro-8-oxoguanine (GO) forms one of the major DNA lesions generated by reactive oxygen species (ROS). The GO can be corrected by GO DNA glycosylases (Ogg), enzymes involved in base excision repair (BER). Unrepaired GO induces mismatched base pairing with adenine (A); as a result, the mismatch causes a point mutation, from G paired with cytosine (C) to thymine (T) paired with adenine (A), during DNA replication. Here, we report the characterization of a putative Ogg from the thermoacidophilic archaeon* Thermoplasma volcanium*. The 204-amino acid sequence of the putative Ogg (TVG_RS00315) shares significant sequence homology with the DNA glycosylases of* Methanocaldococcus jannaschii* (MjaOgg) and* Sulfolobus solfataricus* (SsoOgg). The six histidine-tagged recombinant TVG_RS00315 protein gene was expressed in* Escherichia coli* and purified. The Ogg protein is thermostable, with optimal activity near a pH of 7.5 and a temperature of 60°C. The enzyme displays DNA glycosylase, and apurinic/apyrimidinic (AP) lyase activities on GO/N (where N is A, T, G, or C) mismatch; yet it cannot eliminate U from U/G or T from T/G, as mismatch glycosylase (MIG) can. These results indicate that TvoOgg-encoding* TVG_RS00315* is a member of the Ogg2 family of* T. volcanium*.

## 1. Introduction

Production of reactive oxygen species (ROS), such as hydrogen peroxide, superoxide, and hydroxyl radicals, has been linked to the initiation and progression of cancer [1]. ROS are produced by cellular respiration or inflammatory responses as a consequence of ionizing radiation or owing to environmental exposure to transition metals, chemical oxidants, or free radicals. In general, ROS are eliminated enzymatically or nonenzymatically in normal cells. However, aberrantly functioning cells are often in a state of oxidative stress leading to elevated levels of cellular damage [2].

Among the four DNA bases [guanine (G), adenine (A), thymine (T), and cytosine (C)], G is particularly susceptible to oxidative damage from ROS because of its low redox potential [3]. 8-Oxoguanine (7,8-dihydro-8-oxoguanine; GO) is the most common oxidative product derived from G, and the most prevalent lesion observed in DNA molecules. GO induces a mismatched pairing, GO/A, out of the base pairing rules, and replication of the DNA with GO can result in the misincorporation of A opposite the GO. To prevent a transversion mutation, GO is recognized and excised by the base excision repair (BER) pathway [2, 4].

In* Escherichia coli*, three different enzymes cooperate to handle GO in the genome. MutM can excise GO from double-stranded DNA, MutY can excise adenine (A) from GO-A mismatches, and MutT is an 8-oxo-dGTPase that prevents the incorporation of 8-oxo-dGMP into nascent DNA [5, 6]. In eukarya, processing GO is primarily mediated by Ogg. While both MutM and Ogg are bifunctional enzymes that catalyze N-glycosylase and apurinic/apyrimidinic (AP) lyase activities, MutM is mostly found in bacteria and Ogg is found in eukarya and archaea, except for the atypical bacterial Ogg1 from* Clostridium acetobutylicum* (CacOgg) [7].

Ogg enzymes are divided into three separate families, namely, Ogg1, Ogg2, and archaeal GO glycosylase (AGOG) [8]. The structures of Ogg1,* Homo sapiens* Ogg (hOGG1) [9]; Ogg2,* Methanocaldococcus jannaschii* Ogg (MjaOgg) and* Sulfolobus solfataricus* Ogg (SsoOgg) [8]; and AGOG,* Pyrobaculum aerophilum *Ogg (Pa-AGOG) [10] are already resolved. AGOG is a member of the HhH-GPD DNA glycosylase superfamily [10]. An A domain of AGOG contains very low sequence identity with Ogg1 (i.e., 13–19% identity between Pa-AGOG and hOGG1), and the HhH-GPD motif of AGOG is noncanonical; the GO recognition activity of AGOG is unlike that observed in hOGG1 because of these differences in the HhH-GPD motif [10].

The Ogg1 glycosylase and AP lyase activities are associated through a common motif: a helix-hairpin-helix DNA binding domain, followed by a glycine-proline-rich stretch and an invariant aspartate (HhH-GPD motif) [11]. Ogg1 displays a high selectivity for GO/C pairings, interacts with XRCC1, an essential protein required for the maintenance of genomic stability through DNA repair, and is critical to BER [12].

Ogg2 lacks amino acid residues Asn149, Arg154, and Tyr203 when compared to hOGG1, providing an explanation for Ogg2's reduced specificity, relative to Ogg1, for the base positioned opposite the lesion. The C-terminal lysine of Ogg2 may play a key role in discriminating between G and GO [13]. This prediction was confirmed by measuring the glycosylase/lyase activity of a deletion mutant of MjaOgg lacking the three amino acids at the C-terminal and subsequent cocrystallization of MjaOgg with DNA containing GO sequences [8].

*Thermoplasma volcanium* is known to possess several kinds of DNA repair enzymes. TVG_RS00235 (TVN0046) encodes an AP endonuclease that is a homolog of* E. coli* AP endonuclease (ExoIII) and human protein APE1 [14]. TVG_RS04325 (TVN0804) shares 50% amino acid sequence homology with* Pa*-MIG (PAE3199) of the hyperthermophilic archaeon* Pyrobaculum aerophilum* that eliminates uracil (U) and T from U/G, T/G, U/GO, and T/GO mismatches with an uncoupled AP lyase activity [15]. TVG_RS04465 (TVN0827), a homolog of TA_RS02485 (Ta0477) with 68% amino acid sequence identity, encodes a uracil DNA glycosylase [16]. Its homolog,* Pa*-UDGb, was reported as the fifth uracil DNA glycosylase family member with catalytic activity for the removal of an aberrant purine, hypoxanthine, from DNA [17].

In the process of genomic SELEX experiments using a ferric uptake regulatory protein, TvFur, we found that TvFur binds the promoter region of* TVG_RS00310* (*TVN0061*), which encodes superoxide dismutase, TvSOD [18]. The TvSOD gene is located at the 5′ end of an operon. This operon is composed of two genes:* tvsod* and TVG_RS00315 (TVN0062). TVG_RS00315 (TVN0062) encodes a putative Ogg protein homologous to Ogg2 family members MjaOgg and SsoOgg. That is, these two genes constitute a single transcription unit. This gene configuration makes it possible to rapidly respond to oxidative stress.

In this paper, we cloned the TVG_RS00315 DNA of the thermoacidophilic archaeon* T. volcanium*, encoding a putative TvoOgg protein. TVG_RS00315 protein seemed, by homology, likely to belong to the Ogg2 family. To test this, we expressed the TvoOgg gene in* E. coli*, purified the corresponding protein, and examined its GO glycosylase/lyase activity. The preference for bases positioned opposite GO and other glycosylase activities were also investigated.

## 2. Materials and Methods

### 2.1. Bacterial Strains

*Thermoplasma volcanium* GSS1 (Japan Collection of Microorganisms; JCM 9571), a strain of* Thermoplasma volcanium* isolated from submarine and continental solfataras at Volcano Island, Italy [19], was used for the extraction of* T. volcanium *total genomic DNA.* E. coli* strain JM109 was used for the cloning of the ORF of the putative* TvoOgg*, TVG_RS00315.* E. coli* strain BL21 (DE3) was used as a host strain to obtain TVG_RS00315 protein.

### 2.2. Medium

Luria-Bertani (LB) medium was used for the culture of bacteria bearing plasmids or TVG_RS00315 proteins. If necessary, antibiotics were added at the following concentration: ampicillin, 50 *μ*g/mL; kanamycin, 20 *μ*g/mL; chloramphenicol, 34 *μ*g/mL. For expression of TVG_RS00315, 1 mM isopropyl-*β*-D-thiogalactoside (IPTG) was used when needed.

### 2.3. Plasmid

Plasmid pGEM-T Easy (Promega, USA) was used for the cloning of TVG_RS00315, the putative* TvoOgg* gene. Plasmid pET28a(+) (Merck Millipore, Germany) was used for the expression and purification of TVG_RS00315 protein.

### 2.4. Multiple Alignment of Putative TvoOGG, with MjaOgg and SsoOgg

The amino acid sequences of a putative 8-oxoguanine DNA glycosylase from* T. volcanium* (WP_010916318.1), an 8-oxoguanine DNA glycosylase from* M. Jannaschii* (MjaOgg; Q58134), and an 8-oxoguanine DNA glycosylase of* S. Solfataricus* (SsoOgg; WP_009992328) were selected from NCBI protein database. These three amino acid sequences were multiply aligned using a multiple sequence alignment program Clustal Omega [20].

### 2.5. Cloning, Overexpression, and Purification of TvoOgg

The candidate* TvoOgg* (GenBank Gene ID: 1441548, TVG_RS00315, annotated N-glycosylase) was identified by sequence analysis of the complete genome sequence of* T. volcanium* [21]. The DNA fragment of the ORF of TVG_RS00315 was amplified by PCR using the primers TvoOgg 5′ Nde I (5′-ATC ATA TGG ATT TTA ACC AGT ATT T-3′) and TvoOgg 3′ Sal I (5′-TAG TCG ACT TAC TTT ATA ACT GTC CTT G-3′), which contain* Nde* I and* Sal* I restriction site, respectively (restriction enzyme sites underlined). The PCR with* T. volcanium* genomic DNA as a template was performed for 35 cycles under the following conditions: 94°C for 1 min, 55°C for 1 min, and 72°C for 1 min. The PCR product was cloned into the vector pGEM-T Easy (Promega, USA), amplified in* E. coli* JM109, and extracted by use of a QIAGEN mini-prep kit (QIAGEN, Netherlands). The cloned ORF of TVG_RS00315 DNA was then spliced out by digestion with both* Nde* I and* Sal* I. The DNA fragment corresponding to the ORF of TVG_RS00315 was gel-purified and subcloned into an* Nde* I/*Sal* I-digested pET28a(+) expression vector (Merck Millipore, Germany) to generate pET28a-TvoOgg. The sequence of the cloned ORF of TVG_RS00315 DNA was confirmed by dideoxy sequencing using a CEQ Dye Terminator Cycle Sequencing Quick Start Kit (Beckman Coulter, USA) with T7 promoter primer (5′-TAA TAC GAC TCA CTA TAG-3′) and T7 terminator primer (5′-GCT AGT TAT TGC TCA GCG-3′). Subsequently,* E. coli* expression strain BL21 (DE3) (Merck Millipore, Germany) was transformed with pET28a-TvoOgg.* E. coli* cells harboring pET28a-TvoOgg were grown at 37°C in 200 mL LB medium containing 20 *μ*g/mL kanamycin and 34 *μ*g/mL chloramphenicol to an OD600 of 0.5, and then the expression of the TvoOgg protein with an N-terminal six histidine- (His_6_-) tag was induced, by the addition of IPTG at a final concentration of 1 mM, over 5 hr of incubation. The cells were harvested and suspended in 10 mL of lysis buffer (50 mM NaH_2_PO_4_, 300 mM NaCl, 10 mM imidazole, and 5 mM 2-mercaptoethanol, pH 8.0). Cell suspensions were subjected to sonication (twelve times of 10 sec pulse, with 10 sec intervals at 4 W output with a microtip) with a Sonicator 3000 (WAKEN BTECH, Japan). Cell debris was removed by centrifugation at 8,000 ×g for 10 min at 4°C. The supernatant was applied to a column embedded with 1.5 mL of Ni-NTA agarose (QIAGEN, Netherlands). The column was washed by 10 mL of wash buffer (50 mM NaH_2_PO_4_, 300 mM NaCl, 20 mM imidazole, and 5 mM 2-mercaptoethanol, pH 8.0). TvoOgg protein was then eluted with 10 mL of an elution buffer (50 mM NaH_2_PO_4_, 300 mM NaCl, 200 mM imidazole, and 5 mM 2-mercaptoethanol, pH 8.0) and fractionated into 1 mL aliquots. A portion of each eluted fraction was subjected to a 15% SDS-PAGE analysis. Fractions containing His_6_-tagged TvoOgg were pooled and dialyzed against dialysis buffer 1 (150 mM NaCl, 50 mM KH_2_PO_4_, and 5 mM 2-mercaptoethanol, pH 7.5) at 4°C overnight. Then, the dialyzed TvoOgg protein was applied to a column embedded with 1.5 mL of SP Sepharose FF (GE healthcare, USA) equilibrated with dialysis buffer 1. The column was washed with dialysis buffer 1, and the TvoOgg was eluted with a stepwise gradient comprising 1 mL of 100 mM NaCl, 1 mL of 200 mM NaCl, 2 mL of 250 mM NaCl, 1 mL of 300 mM NaCl, and 5 mL of 400 mM NaCl in dialysis buffer 1. Eluted fractions were subjected to a 15% SDS-PAGE analysis, and fractions containing TvoOgg were pooled and dialyzed against dialysis buffer 2 (150 mM NaCl, 5 mM 2-mercaptoethanol, and 50 mM Tris-HCl, pH 7.5). Dialyzed fractions containing TvoOgg were concentrated with a Centricon centrifugal filter device YM-10 (Merck Millipore, Germany) and stored in dialysis buffer 2 at −80°C until use. Protein concentration was determined by measurement of absorbance at 280 nm.

### 2.6. Oligonucleotide Substrates

The 34-mer oligonucleotides, oligo1, 5′-TGT CAA TAG CAA G**X**G GAG AAG TCA ATC GTA GTC T-3′, and oligo2, 5′-AGA CTA CGA TTG ACT TCT CC**Y** CTT GCT ATT GAC A-3′, were synthesized (Merck Millipore, Germany). In variants of each oligonucleotide,** X** or** Y** corresponded to A, T, G, C, U, or 7,8-dihydro-8-oxoguanine (GO). For the detection of strand breaks, the 5′ end of the oligonucleotides was labeled with a fluorescent dye, FAM. Oligo1 and oligo2 were annealed to form double-stranded DNA containing the base pair** X**/**Y** at position 14. This double-stranded 34-bp DNA was used for substrates for glycosylase and/or AP lyase activity assays.

### 2.7. Glycosylase Activity Assay

In the experiments described below, 1 pmol (50 nM) of TvoOgg was used for the DNA glycosylase and/or AP lyase activity assays, and the reactions were carried out at 60°C for 30 min, unless otherwise mentioned. The glycosylase activity assay was carried out according to Yang et al. [15]. The glycosylase activity was detected by the combined action of the glycosylase and the subsequent cleavage of DNA at AP site. The reaction mixture contained 1 mM dithiothreitol (DTT), 1 mM EDTA, 3% glycerol, 80 mM NaCl, 20 mM Tris-HCl, and 4 pmol of labeled, double-stranded DNA in a total volume of 20 *μ*L at pH 7.5. One pmol of TvoOgg protein was added to the reaction mixture and incubated at 37°C, 50°C, 60°C, 70°C, 80°C, or 90°C for 30 min. The reaction products were treated with 4 *μ*L of 1 M NaOH and heated at 96°C for 4 min to complete cleavage of DNA at AP site before electrophoresis. The reaction mixtures were diluted up to 100 *μ*L with 1 mM EDTA, 10 mM Tris-HCl pH 8.0 (TE) buffer, and DNA was extracted with a phenol-chloroform process. Resultant DNA was suspended in 10 *μ*L of gel loading buffer (95% formamide, 20 mM EDTA, and 5% bromophenol blue) at 94°C for 3 min and then analyzed on 15% polyacrylamide-7 M urea gels. The DNA bands were detected and analyzed with a Pharos FX molecular imager and Quantity One imaging software (Bio-Rad, USA). The dissociation constant (*K*_m_) was calculated according to the method of Castaing et al. [22].

### 2.8. AP Lyase Activity Assay

The preparation of double-stranded DNA containing AP sites was essentially according to the method of Horst and Fritz [23]. Ten pmol of double-stranded DNA containing a U/C mismatch was incubated with 1.25 U of* E. coli* uracil DNA glycosylase (UDG, New England BioLabs, UK), in the reaction buffer for UDG, for 30 min at 37°C. The AP/C substrate was incubated with 1 pmol of TvoOgg, in the same reaction buffer used for the glycosylase activity assay described above, for 30 min at 37°C, 50°C, 60°C, 70°C, 80°C, or 90°C without subsequent alkaline treatment. The reaction products were suspended in 10 *μ*L of gel loading buffer at 94°C for 3 min and then analyzed on 15% polyacrylamide-7 M urea gels.

## 3. Results and Discussion

### 3.1. Homology between Putative TvoOgg Protein and Other OGG2 DNA Glycosylases

**Figure 1:**
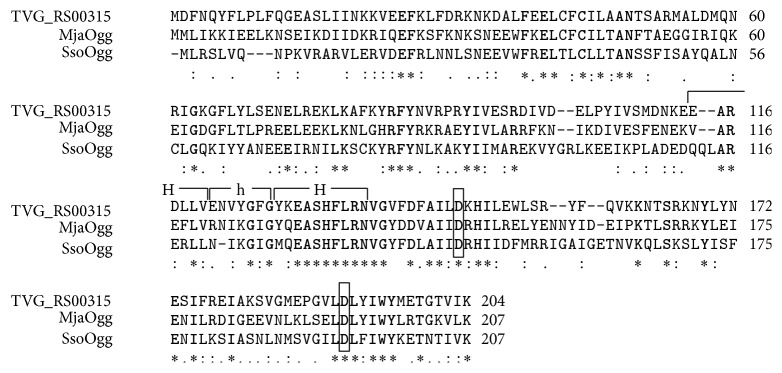
Amino acid sequence alignments of the TVG_RS00315 protein (TvoOgg; WP_010916318.1) with 8-oxoguanine DNA glycosylase of* Methanocaldococcus jannaschii* (MjaOgg; Q58134) and 8-oxoguanine DNA glycosylase of* Sulfolobus solfataricus* (SsoOgg; WP_009992328). The amino acid residues in bold are conserved between the three proteins. “H” and “h” indicate alpha helices and hairpin structures, respectively. The catalytic residue of a conserved aspartate is boxed. Asterisks, colons, and dots indicate positions which have fully conserved, strongly similar, and weakly similar residues, respectively.

The open reading frame (ORF) of TVG_RS00315 (TVN0062) from thermophilic archaeon* T. volcanium*, encoding 204 amino acid residues, was identified through a genome sequencing project [21].  Figure 1 shows the amino acid alignments of the TVG_RS00315 protein (WP_010916318.1) with MjaOgg and SsoOgg. The ORF of TVG_RS00315 was homologous to MjaOgg (207 amino acid residues) with 47% (93/198) identity, and to SsoOgg (207 amino acid residues) with 40% (78/193) identity. The ORF of TVG_RS00315 contains a helix-hairpin-helix motif. The catalytic lysine (Lys129 in MjaOgg and Lys128 in SsoOgg) and aspartate (Asp147 in MjaOgg and Asp146 in SsoOgg) are conserved in TVG_RS00315 at Lys129 and Asp147, respectively. At Lys204, the ORF of TVG_RS00315 also possessed a C-terminal lysine (Lys207 in MjaOgg and Lys207 in SsoOgg) that discriminates between G and GO. In MjaOgg, Phe85 is wedged between the estranged cytosine and its 5′-neighbor, His133 and Trp198 sandwich the aberrant GO, and Arg84 interacts with the estranged cytosine [13]; all of these crucial residues were conserved in the putative TvoOgg, TVG_RS00315 (Figure 1). These results strongly suggested that the ORF of TVG_RS00315, TvoOgg, is a homolog of Ogg2. In order to determine if the protein encoded by* TVG_RS00315* serves as GO glycosylase, we cloned and expressed the putative TvoOgg and investigated the function of this protein.

### 3.2. Production of TvoOgg Protein in* E. coli* and Purification of the Recombinant Protein

**Figure 2:**
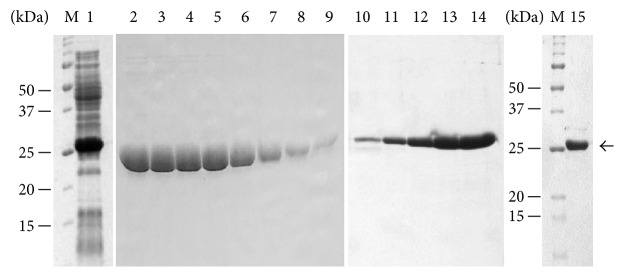
Purification of TvoOgg recombinant protein from* E. coli*. A Coomassie brilliant blue stained SDS-polyacrylamide gel (15%) showing separation of a lysate of BL21 (DE3) cells containing pET28a-TvoOgg after induction by 1 mM IPTG for 5 hr (lane 1), eluted fractions of TvoOgg from Ni-NTA agarose columns (lane 2–9), or SP Sepharose FF columns (lane 10–14), respectively, and TvoOgg protein after dialysis against dialysis buffer 1 (lane 15). The TvoOgg protein is indicated by an arrow. Lane M contained molecular mass standards (Bio-Rad, U.S.A.) as indicated on the left.

The DNA encoding the TvoOgg was cloned into pGEM-T Easy vector, spliced out after digestion with* Nde* I and* Sal* I, subcloned into* Nde* I/*Sal* I double digested pET28a(+) behind a His_6_-tag, and expressed in* E. coli* BL21(DE3) (Figure 2, lane 1). The His_6_-tagged TvoOgg was purified from the cell lysate by a heat treatment of 60°C for 30 min, subjected to Ni^2+^-NTA column chromatography (Figure 2, lane 2–9), and then to SP Sepharose FF column chromatography (Figure 2, lane 10–14) to produce essentially pure protein. The TvoOgg is heat-stable up to at least 60°, and highly soluble (data not shown). The approximate molecular mass of the purified protein on SDS-PAGE was slightly larger than 25 kDa (Figure 2, lane 15). This value was consistent with the molecular mass of 26.4 kDa predicted from the His_6_-tagged TvoOgg amino acid sequence.

### 3.3. Temperature Optimum of Glycosylase and Lyase Activity of TvoOgg

**Figure 3:**
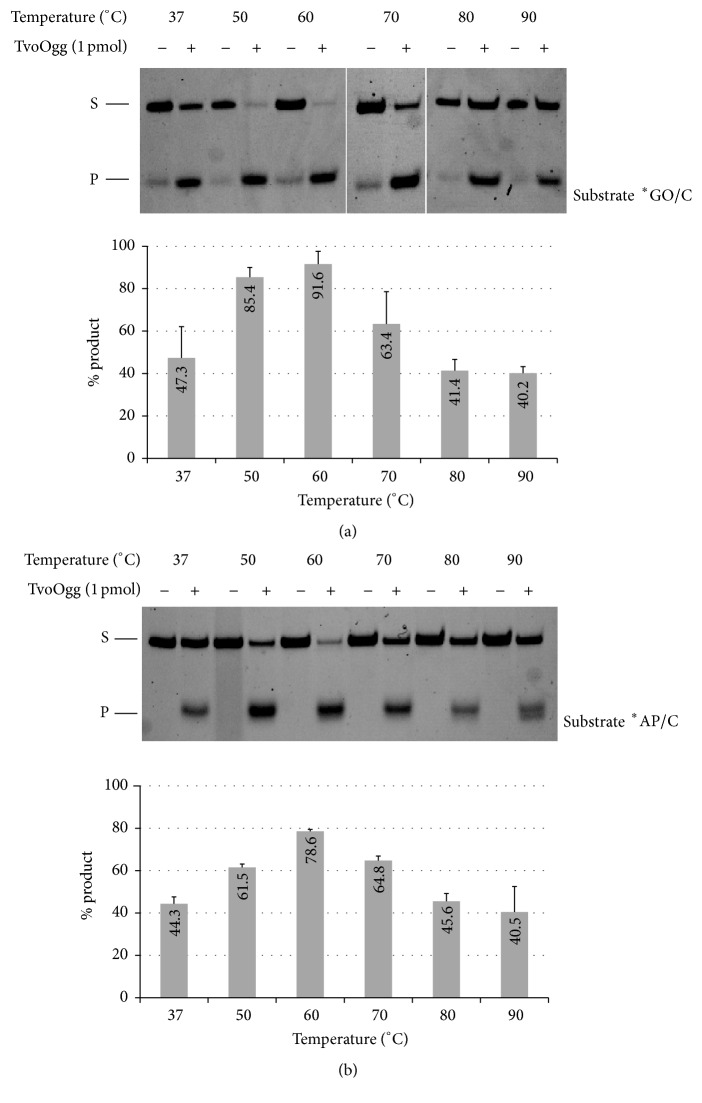
Temperature dependency of GO glycosylase activity (a), or AP lyase activity (b), in the presence (+) or absence (−) of 1 pmol of TvoOgg with a 34-bp heteroduplex DNA containing GO/C (a) or AP/C (b), respectively, mismatched at different temperatures for 30 min. The strand containing GO and AP was 5′-end labeled with FAM. The uncut 34-mer DNA substrate (S) and cleaved 13-mer product (P) were indicated on the left. The signal intensities of each product were measured and quantified with Pharos FX. Data are presented as the mean ± SD of three independent measurements. Asterisks indicate the 5′-end labeled strand.

Since MutM and Ogg family members are known as bifunctional glycosylases, we tested glycosylase and lyase activities independently using GO/C double-stranded DNA. GO DNA glycosylase and AP lyase activity assays on TvoOgg were carried out at 37°C, 50°C, 60°C, 70°C, 80°C, or 90°C. GO DNA glycosylase activity of TvoOgg was 1.9-fold or 2.6-fold more active at 60°C (95.0%) than at 37°C (59.8%) or 90°C (36.6%), respectively (Figure 3(a)). At 50°C and 60°C, most of the substrates were converted to the products. The TvoOgg enzyme was slightly active at both 37°C and 90°C. When an AP site containing double-stranded DNA was used for an AP lyase activity assay, nicking products were observed at all temperatures tested. The greatest activity of AP lyase of TvoOgg was observed at 60°C, the same as for the glycosylase activity (Figure 3(b)). However, in contrast to glycosylase activity, the AP lyase exhibited almost the same activity at 50°C and 70°C (Figure 3(b)). Most of the substrates were converted to the products in both the AP lyase assay and the NaOH-treated GO glycosylase assay at 60°C (Figures 3(a) and 3(b)). This indicated that the GO glycosylase and AP lyase activity of TvoOgg have similar temperature and pH optimum. The reduction of activity of TvoOgg at temperatures higher than 60°C (the optimal growth temperature of* T. volcanium*) was probably due to the heat instability of the enzyme or instability of the double-stranded DNA substrate at high temperature.

### 3.4. Time Course of Glycosylase Activity of TvoOgg Protein

**Figure 4:**
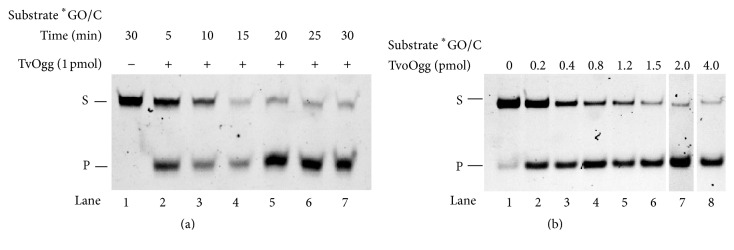
GO glycosylase/lyase activity assay of putative TvoOgg. (a) Time course of TvoOgg glycosylase activity on substrate ^*∗*^GO/C. 34-bp heteroduplex DNA containing a ^*∗*^GO/C mismatch was incubated with (lanes 2 to 7) or without (lane 1) 1 pmol of TvoOgg. The uncut 34-mer DNA substrate (S) and cleaved 13-mer product (P) are indicated on the left. (b) Dose dependency of ^*∗*^GO/C mismatch-specific DNA glycosylase activity of TvoOgg on 34-bp double-stranded DNA containing GO/C. The uncut 34-mer DNA substrates (S) and cleaved 13-mer products (P) are indicated on the left. Asterisk indicated the 5′FAM-labeled strand.

The GO-containing strand was FAM-labeled at the 5′ end and annealed with a complementary oligonucleotide sequence possessing C positioned opposite the GO base. This double-stranded DNA was used for a substrate for GO DNA glycosylase and AP lyase activity assay of TvoOgg. The candidate of TvoOgg protein catalyzed the removal of GO from the GO/C double-stranded DNA substrate (Figures 4(a) and 4(b)). The cleavage reaction of the DNA was saturated by 20 or 25 min of reaction at 60°C. This result indicated that TvoOgg is a TvoOgg that has GO DNA glycosylase activity. This GO DNA glycosylase activity was dose-dependent (Figure 4(b)). When 0.4 pmol (20 nM) of TvoOgg protein was used for the reaction, approximately equal amounts of the substrate and the product were observed (Figure 4(a)). From this result, the approximate *K*_m_ of TvoOgg was 20 nM at 60°C.

### 3.5. Substrate Preference of TvoOgg

**Figure 5:**
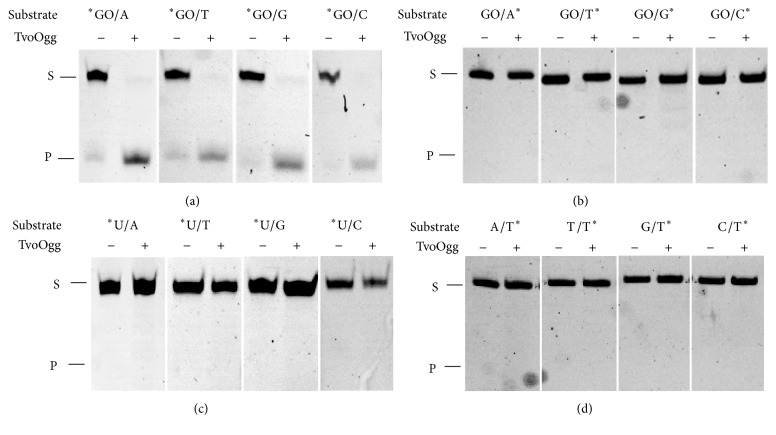
Requirement of GO for the substrates of TvoOgg. TvoOgg glycosylase/lyase activity was determined by analysis of the products in the presence (+) or absence (−) of 50 nM of TvoOgg on 34-bp double-stranded DNA containing (a) ^*∗*^GO/N, (b) GO/N^*∗*^, (c) ^*∗*^U/N, or (d) A/N^*∗*^ (N means A, T, G, or C). The uncut 34-mer DNA substrates (S) and cleaved 13-mer products (P) are indicated on the left. Asterisks indicate 5′-FAM-labeled strand.

**Figure 6:**
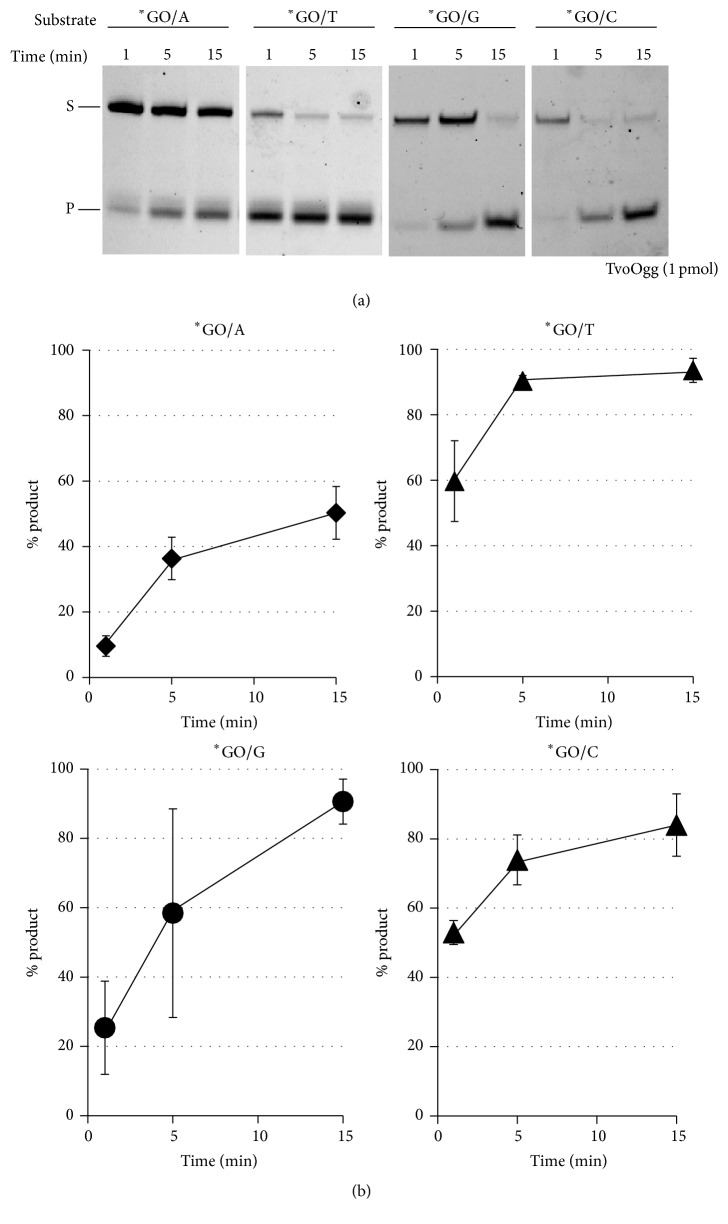
Time course of TvoOgg activity on substrates ^*∗*^GO/A, ^*∗*^GO/T, ^*∗*^GO/G, and ^*∗*^GO/C. One pmol of TvoOgg was incubated with heteroduplex DNA containing ^*∗*^GO/A, ^*∗*^GO/T, ^*∗*^GO/G, and ^*∗*^GO/C mismatches at 60°C. At the indicated time points, the reaction was quenched with 4 *μ*L of 1 M NaOH to inactivate enzyme and cleave resulting AP sites. The uncut 34-mer DNA substrates (S) and cleaved 13-mer products (P) are indicated on the left. Asterisks indicate 5′-FAM-labeled strand. Each data point is represented as the mean ± SD of three independent measurements.

To determine whether the base in the complementary strand of GO is critical for the base excision activity of TvoOgg, we conducted DNA glycosylase activity assays using double-stranded DNA substrates containing GO/N, U/N, or T/N (N means A, T, G, or C). When the GO/N substrates were labeled at the 5′ end of the DNA strand containing GO, TvoOgg could efficiently excise GO from GO/N-containing substrates. This enzyme could excise GO positioned opposite any bases (Figure 5(a)). The cleavages were detected only for the DNA strands containing GO. TvoOgg could eliminate the GO from GO/N pairings but could not eliminate N from those pairings (N means A, T, G, and C, Figure 5(b)). TvoOgg exhibited no uracil DNA glycosylase activity for any of the four DNA bases positioned opposite U mismatches (Figure 5(c)) and could not exhibit base excision activity on the T:A base pair or T mispaired with C, T, or G (Figure 5(d)). These results indicate that the DNA glycosylase activity of TvoOgg protein is not capable of acting upon double-stranded DNA substrates containing U/N or T/N, which differs from Mth-MIG and* Pa*-MIG mismatch glycosylases [15]. We carried out time course experiments with four substrates, GO/N labeled at the 5′ end of the DNA strand containing GO to confirm the opposite base specificity of the glycosylase activity of TvoOgg. As shown in Figure 6, TvoOgg preferred substrate DNA duplexes containing GO/C, GO/T, and GO/G but had the poorest activity on GO/A (Figure 6). This substrate preference of TvoOgg is similar to Afogg [24] but different from MjaOgg [8].

In this study, we characterized an ORF of a candidate of GO DNA glycosylase/lyase in the thermoacidophilic archaeon,* T. volcanium, *as a TvoOgg. The validity of this definition was also supported by the glycosylase/lyase activity of this protein for GO/N mismatches (Figure 5).

AP endonuclease of* T. volcanium*, TVG_RS00235 (TVN0046), was cloned and characterized by Kaneda et al. [14]. This enzyme recognizes the AP site in DNA and then cleaves on the 5′ side of AP site through its AP endonuclease activity. TVG_RS00235 (TVN0046) also shows 3′-5′ exonuclease activity. Two tryptophan residues (Trp200 and Trp214) contribute to its AP site recognition [14].

*Pa*-MIG contains a 4Fe-4S binding site in its C-terminal portion and is a U/G and T/G mismatches-specific glycosylase.* Pa*-MIG eliminates T and U positioned opposite GO or G [15]. TVG_RS04325 (TVN0804) protein is a homolog of* Pa*-MIG, and its base specificity is different from that of the TvoOgg (which could not recognize U positioned opposite G, or T positioned opposite G or GO) (Figure 5). The enzyme encoded by TVG_RS04325 (TVN0804) may contribute to the elimination of U/G and T/G mismatches by hydrolytic deamination of cytosine or 5-methylcytosine in the double-stranded DNA of* T. volcanium*. TVG_RS04325 (TVN0804) protein might play the same function of* Pa*-MIG, to prevent G to A substitutions by engendering the hydrolytic deamination of C or methyl-C.

TVG_RS04465 (TVN0827) protein was reported as one of six family members of the* Pa*-UDGb family, the fifth uracil DNA glycosylase family, according to Sartori et al. [17].* Pa*-UDGb is a uracil DNA glycosylase with broad substrate specificity because of the lack of an essential polar residue in motif A of its active site. This enzyme efficiently catalyzes the removal of uracil, hydroxymethyluracil, or ethenocytosine that is positioned opposite G, without AP lyase activity, and is less effective on the bases positioned opposite A. It can, however, excise an aberrant purine, hypoxanthine, from mispairing with T [17].

*Archaeoglobus fulgidus* Ogg (Afogg), MjaOgg, and SsoOgg all belong in the Ogg2 family of GO DNA glycosylases coupled with AP lyase activity. Afogg protein efficiently cleaves DNA containing GO/C and GO/G base pairs but is less effective on DNA containing GO/T or GO/A mispairings [24]. From this substrate specificity, Afogg prevents G/C to T/A transversion mutations from GO/C mispairing by eliminating GO but cannot eliminate mismatched A from GO/A. This substrate specificity is similar to that of TvoOgg. MjaOgg indicated different base preference from Afogg and TvoOgg; MjaOgg protein efficiently cleaves DNA containing GO across from any of the four canonical bases [8]. Since the BER activity of recombinant SsoOgg protein has not been reported, its base preference for bases positioned opposite GO are not known. TvoOgg could excise GO positioned opposite all four bases (Figures 5 and 6), indicating that this enzyme is robust at preventing GO/A mispairings that, if left uncorrected, generate C/G to T/A transversion mutations during the next round of replication.

*Pa*-AGOG bears an HhH-GPD motif and can remove GO from GO/G, GO/T, or GO-containing single-stranded DNA effectively; however,* Pa*-AGOG displays weak processing activity of GO/C or GO/A [10]. In the* T. volcanium* genome, a homolog of* Pa*-AGOG is undetectable by either a conventional sequence search or short-sequence motif search (data not show). This result indicates that* T. volcanium* does not possess AGOG family GO DNA glycosylases like* P. aerophilum* and that Ogg2 is responsible for eliminating 8-oxoguanine from DNA in* T. volcanium*.

Unlike* M. jannaschii* and* A. fulgidus*,* T. volcanium* can be grown under aerobic condition. Because 8-oxoG results from the oxidation of guanine in DNA via oxidizing agents or ROS, it is the most abundant oxidative lesion in DNA for organisms that live in aerobic environments. The TvoOgg gene is located at the 3′ end of an operon, composed of two genes,* tvsod* and* tvoogg*.* T. volcanium* may effectively avoid the transversion mutation generated by oxidative stress by this gene organization.

## 4. Conclusion

The open reading frame of TVG_RS00315 (TVN0062) from the thermoacidophilic archaeon* Thermoplasma volcanium* (TvoOgg) belonging to the Ogg2 family was overexpressed in* E. coli* and biochemically characterized. TvoOgg preferred GO/N as its most active substrate, but its activities toward U/N or A/N substrates were hard to measure. In this study, we found that the optimal temperature for TvoOgg activity is 60°C. The characteristic of possessing GO glycosylase/lyase activity of TvoOgg at a relatively low temperature (50°C) should play an important role in the survival of* T. volcanium* under low temperature conditions.

*T. volcanium* possesses several BER enzymes. TVG_RS04325 (TVN0804) is a homolog of Pa-MIG that eliminates T from GO/T and G/T and U from GO/U and G/U. TVG_RS00235 (TVN0046) is an AP endonuclease that recognizes AP sites in DNA. TVG_RS04465 (TVN0827) is reported as a* Pa*-UDGb, a uracil DNA glycosylase with broad substrate specificity. A homolog of* Pa*-AGOG is not encoded in the* T. volcanium* genome. TVG_RS00315 protein could excise GO positioned opposite all four bases. These results indicate that TVG_RS00315 protein is a TvoOgg and robustly prevents GO/A mispairings that generate C/G to T/A transversion mutations during a round of replication.* TvoOgg* is located tandemly with a superoxide dismutase gene,* tvsod*. This gene organization of* T. volcanium* may cancel out the oxidative stress inherent in living under aerobic conditions.
